# YTHDC2-Mediated circYTHDC2 N6-Methyladenosine Modification Promotes Vascular Smooth Muscle Cells Dysfunction Through Inhibiting Ten-Eleven Translocation 2

**DOI:** 10.3389/fcvm.2021.686293

**Published:** 2021-10-01

**Authors:** Jun Yuan, Yu Liu, Lizhen Zhou, Yan Xue, Zhengde Lu, Jianting Gan

**Affiliations:** ^1^Department of Cardiology, The People's Hospital of Guangxi Zhuang Autonomous Region, Nanning, China; ^2^Health Management Center, The People's Hospital of Guangxi Zhuang Autonomous Region, Nanning, China

**Keywords:** vascular smooth muscle cells, circular RNAs, N6-methyladenosine, hyperglycemia, ten-eleven translocation 2 (TET2)

## Abstract

Type 2 diabetes condition mediated vascular smooth muscle cell (VSMCs) dysfunction. However, the mechanism of VSMCs dysfunction in diabetic patients needs further elucidation. VSMCs are an important component of the vascular wall, participate in the process of vascular remodeling, and play a vital role in the vascular complications of diabetes. Studies have found that circular RNAs (circRNAs) play a key regulatory role in the occurrence and development of VSMCs dysfunction. In this study, we stimulated VSMCs with high glucose and identified a new circular RNA, circYTHDC2, using circRNA chip analysis. circYTHDC2 was highly expressed in VSMCs treated with high glucose. Knockout of circYTHDC2 significantly inhibited the proliferation and migration of VSMCs. Metformin treatment significantly inhibited the expression of YTHDC2 and circYTHDC2. The upstream mechanism analysis revealed that the stability of circYTHDC2 was regulated by YTHDC2-mediated m6A modification. Furthermore, circYTHDC2 negatively regulates the expression of Ten-Eleven Translocation 2 (TET2) by targeting the unstable motif of TET2 3′UTR, thereby promoting dedifferentiated “synthetic type” transformation of VSMC. Taken together, these results suggest that the YTHDC2/circYTHDC2/TET2 pathway is an important target of metformin in preventing the progression of VSMCs dysfunction under high glucose.

## Introduction

Type 2 diabetes (T2DM) is one of the most serious public health problems in the world (1). In recent years, the mortality rate of diabetes combined with coronary heart disease has been increasing year by year. Sixty percentage to 70% of diabetic patients will eventually die of cardiovascular disease (2). Vascular smooth muscle cell (VSMCs) dysfunction played an important role in T2DM-mediated cardiovascular disease (3). However, the formation mechanism of VSMCs dysfunction in diabetic patients needs further elucidation.

VSMCs are an important component of the vascular wall, participate in the process of vascular remodeling, and play a vital role in the vascular complications of diabetes (4). High concentration of glucose promotes VSMC proliferation and DNA synthesis (5). The proliferation and migration of VSMC enhances the neointimal hyperplasia, the elastic retraction of the adventitia and the decrease of the inner diameter of the vascular lumen, which are important factors that promote the occurrence and development of cardiovascular disease (6).

Circular RNAs (circRNAs) are a special kind of non-coding endogenous RNAs (non-coding endogenous RNA, neRNA), which are abundantly present in the transcriptome of eukaryotic cells (7). The main difference between circRNA and linear RNA is that the former has no 5′ terminal and 3′ terminal polyadenylic acid tails, which are closed circular RNA molecules formed by covalent bonds. The special structure of circRNA makes it difficult to be hydrolyzed by exonuclease, so it is more stable than linear RNA and has evolutionary conservation. In recent years, many studies have found that circRNAs play a key regulatory role in the occurrence and development of cardiovascular disease (8). For example, overexpression of circular RNA circ-SATB2 promotes the proliferation and migration of VSMCs and inhibits cell apoptosis (9). Knockdown circCHFR can effectively inhibit the proliferation and migration of VSMCs *in vitro* (10). The above results indicate that circRNA can regulate the functional changes of VSMCs, and provide new ideas for the diagnosis and treatment of cardiovascular diseases. However, the upstream regulatory mechanism of circRNAs in the progression of VSMCs dysfunction is still unclear.

Recent studies have found that the production and function of circRNA are regulated by N6-methyladenosine (m6A) modification (11). m6A refers to the methylation modification that occurs on the 6th nitrogen atom of adenine, which is widely present in the RNA of many eukaryotes. The current researches find that m6A is a dynamic process, and its function is jointly determined by the “Writer,” “Eraser,” and “Reader” (12). The function of m6A is recognized by the “reader,” that is m6A binding protein. The m6A binding proteins so far include YTH (YT521-B homology) domain protein family, namely YTHDF1, YTHDF2, YTHDF3, YTHDC1, YTHDC2 and the HNRNP family of nuclear heterogeneous proteins, namely HNRNPA2B1 and HNRNPC (13). The evolutionarily conserved YTH domain contained in the YTH protein family can selectively recognize m6A. YTHDC2 contains a YTH domain at the C-terminus, which mediates the binding to m6A. The interaction with 5′-3′ exonuclease XRN1 indicates that YTHDC2 may play a role in regulating mRNA stability (14). Studies have shown that the recognition of m6A by YTHDC2 is essential for embryonic differentiation. YTHDC2 is highly expressed in the testis of mice, and mice that knock out ythdc2 will develop obstacles in germ cell development and cause infertility (15). YTHDC2 may also be associated with autism, pancreatic cancer, and liver cancer (16–18). However, the relationship between YTHDC2 and VSMCs dysfunction is rarely studied.

In this study, we identified a new circular RNA, circYTHDC2. circYTHDC2 was highly expressed in VSMCs treated with high glucose. Knockout of circYTHDC2 significantly inhibited the proliferation and migration of VSMCs. Metformin treatment inhibited the expression of YTHDC2 and circYTHDC2. The mechanism analysis revealed that YTHDC2-mediated m6A modification stabilized circYTHDC2. In addition, circYTHDC2 negatively regulated the expression of Ten-Eleven Translocation 2 (TET2) by targeting the unstable motif of TET2 3′UTR, thereby promoting the proliferation and migration of VSMCs. These findings suggest that the YTHDC2/circYTHDC2/TET2 pathway is an important target of metformin in preventing the progression of VSMCs dysfunction.

## Materials and Methods

### Culture of VSMCs

The VSMC cell line A7R5 was purchased from the Cell Bank of the Chinese Academy of Sciences (Shanghai, China). VSMCs were cultured in DMEM medium containing 10% fetal bovine serum, cultured in a cell incubator at 37°C and 5% CO2, and 3–5 generations of cells in the logarithmic phase were used for experiments. For VSMCs stimulated by high glucose, 25 mmol/L glucose (high glucose group) was added and cultured for 24 h for subsequent experiments. VSMCs cultured with 5 mmol/L glucose were used as a negative control (19, 20).

### Isolation of Primary VSMC

Animal experiments were approved by the Ethics Committee for Animal Research of The People's Hospital of Guangxi Zhuang Autonomous Region. Sprague Dawley rats were anesthetized by intraperitoneal injection of 1.5% pentobarbital sodium at 0.2 mL/100 g. The thoracic aorta was separated under aseptic conditions, and was placed in pre-cooled DMEM medium, and the tunica media was separated under a microscope. The tunica media were cut into small pieces of 0.5–1 mm^2^, and digested with type II collagenase (2 mg/mL) in a 37°C water bath. The cells were collected by centrifugation, and resuspended in DMEM medium containing 20% FBS. The collected cells were planted in a 6 cm polylysine-coated culture dish, and were cultured in cell incubator for 48 h. The cells were sub-cultured in DMEM medium containing 10% FBS after fusion to 80%. For primary VSMCs stimulated by high glucose, 25 mmol/L glucose (high glucose group) was added and cultured for 24 h for subsequent experiments. primary VSMCs cultured with 5 mmol/L glucose were used as a negative control.

### CircRNA Array Analysis

The Arraystar Human circRNA Microarray was used in this study to analyze the expression of human circRNA globally. About 5,816 circRNAs can be detected by this chip. The sample labeling and chip hybridization were performed according to the Agilent One Color Microarray Based Gene Expression Analysis protocol (Agilent Technologies, Santa Clara, CA, USA). mRNA was isolated from VSMCs stimulated by high glucose (25 mmol/L). VSMCs cultured with 5 mmol/L glucose were used as a negative control. A mRNA ONLY Eukaryotic mRNA Isolation Kit (Epicenter Technologies Corp., Chicago, IL, USA) was used to purify RNA. Then random primers were used to enrich each sample and transcribe it into fluorescent cRNA without 3′offset. The labeled cRNA was purified using RNeasy Mini Kit (Qiagen, Germany), and then the concentration and activity were detected using NanoDrop ND-1000 (Thermo Fisher Scientific Inc., Waltham, MA, USA). Add 5/μl 10x blocking solution and one 25x lysis buffer to each labeled cRNA (1 μg), then heat at 60°C for 30 min, and then add 25xGE hybridization buffer. Pour the hybridized liquid (50 μl in total) on the padded glass slide. Incubate for 17 h at 65°C in an Agilent hybrid incubator. Finally, the hybridization chip was cleaned, fixed, and scanned with Agilent DNA Microarray Scanner (G2505C). Agilent Feature Extraction software (version 11.0.1.1) was used to analyze the obtained chip image. The Greenspring GX v1.5.1 software package (Agilent Technologies, Santa Clam, CA, USA) was used for quantile standardization and subsequent data processing. After quantile standardization of the raw data, the mRNAs with the defined value (all target values) were selected for further data analysis. Agilent Greenspring GX software (version l 1.5.1) was used to make volcano map.

### Actinomycin D and RNase R Treatment

VSMCs were implanted in a six-well plate and cultured to a confluence of 70%. The cells were treated with 5 μg/ml actinomycin D and collected at the specified time point. Total RNA (2 μg) and 3 U/μg RNase R (Epicenter Technologies, Madison, WI, USA) were mixed and incubated at 37°C for 15 min. After treatment with actinomycin D or RNase R, the RNA expression levels of circYTHDC2 and YTHDC2 were analyzed by qRT-PCR.

### Real-Time Quantitative PCR

QPCR was used to detect the expression levels of circRNA and mRNA. After indicated treatment, cells were collected and lysed in 1 mL of Trizol at room temperature for 10 min. The total RNA was extracted by chloroform, isopropanol, and 75% ethanol. The RNA was dissolved in 30 μl DEPC water, and used to perform reverse transcription according to the instructions of the TaKaRa Reverse Transcription Kit. QPCR test was performed according to the instructions of the SYBR Premix Ex Taq kit (cat no. RR820A, TAKARA, Dalian, China). The reaction conditions of qPCR are as follows: 95.0°C for 3 min, and 39 circles of 95.0°C for 10 s and 60°C for 30 s. Data were processed using 2^−ΔΔCT^ method. GAPDH was used as the internal reference. The primers are shown in Supplementary Table 1.

### RNA Fluorescence *in situ* Hybridization

The sequence of the oligonucleotide modification probe circYTHDC2 (circYTHDC2 probe sequence: 5′-Cy5-AAAGACCACACAGAAAGGGAGCAAA-3′) was synthesized by Guangzhou Ribo Biological Company (Guangzhou, China). VSMCs cells were fixed with paraformaldehyde for 12 h and then treated with RNase R for 15 min at 37°C. After dehydration with ethanol, the cells were hybridized with the probe overnight at 37°C under dark conditions. The cells were washed twice with 50% formamide for 5 min each, and then incubated with Cy5 fluorescent secondary antibody (cat no. ab97172, Abcam, USA) for 30 min, and sealed with a parafilm containing DAPI. The pictures were captured using a fluorescence microscope (OLYMPUS, Japan).

### Lentiviral Transfection

The siRNA sequences used to knock out circYTHDC2, YTHDC2 and TET2 and the lentiviral particles overexpressing circYTHDC2 and TET2 were designed and packaged by Guangzhou Ribo Biological Company (Guangzhou, China). The siRNA sequence is: circYTHDC2 siRNA 1#, ACCACACAGAAAGGGAGCAAA; circYTHDC2 siRNA 2#, GACCACACAGAAAGGGAGCAA; YTHDC2 siRNA, CCGGGTGAACCTCTTCATAAGATATCTCGAGATATCTTATGAAGAGGTTCACTTTTTG; TET2 siRNA, CCGGCCTCAGAGATATTGTGGGTTTCTCGAGAA ACCCACAATATCTCTGAGGTTTTTTG. VSMC cells were seeded into a 24-well culture plate at a density of 4 × 10^4^ cells per well to ensure that the cell confluence reached about 80% during transfection. The old medium was removed, and 500 μl new complete medium was added. Each well was added with 6 μl of the lentiviral particle stock solution and negative control virus to the 24-well culture plate, and add 0.5 μl polybrene (final concentration 5 ng/m1). The multiplicity of Infection value of lentivirus infection was 50. After 48 h of infection, cells were collected for subsequent experiments.

### CCK-8 Assay

After transfection, the VSMC cells were inoculated into a 96-well plate at 10^4^ cells per well under high glucose. After 24 culture, the medium was refreshed and CCK-8 (K1018, ApexBio Technology, Shanghai, China) was added into the medium. After 1 h incubation in the cell incubator, the absorbance of the sample at the wavelength of 450 nm is detected by the automatic microplate reader. The cell viability calculation was standardized with the scramble treatment group.

### Transwell Assay

A Cultrex 24 Well Collagen IV Cell Invasion Assay kit (cat no. 3458-024-K, R&D Systems, Inc. Minneapolis, MN, USA) was used to determine the invasive ability of VSMCs. Briefly, the transfected VSMCs were cultured in a cell incubator to reached 70–80% confluence under high glucose. The cells were starved in serum-free media for 24 h prior to beginning assay. The cells were harvested and suspended at 1 × 10^6^ cells/ml in serum free media. One hundred microliter of cells per well were add to top chamber and 500 μl of medium per well was added to bottom chamber. Chambers were incubated at 37°C in a CO_2_ incubator for 48 h. After carefully aspirating top chamber and bottom chamber, each well was wash with 100 μl of 1X Wash Buffer. Five-hundred microliter of Cell Dissociation Solution/Calcein-AM was added to bottom chamber, assemble cell invasion chambers, and incubate at 37°C in CO_2_ incubator for 1 h. A PARADIGM Detection Platform (Beckman, Brea, CA) was used to read dissolved Calcein-AM at 485 nm excitation, 520 nm emission.

### Cell Cycle Analysis

Cell Cycle Staining Kit (cat no. KTA2020, Abbkine, Wuhan, China) was used to determine the cell cycle of VSMCs under high glucose. Briefly, cells from each group were trypsinized and washed with cold PBS. The cells were incubated in 70% alcohol at −20°C for 12 h. After washed with cold PBS for three tines, the cells were resuspended in 0.5 mL Staining Solution for 30 min at 37°C protected from light. The cell cycle was analyzed by using BD LSRFortessa (Becton, Dickinson and Company). This assay was repeated 3 independent times.

### Cell Apoptosis Analysis

The cells were digested and collected after indicated treatment. The cells then were resuspended with 50 μl of pre-cold PBS. A Annexin V-FITC Apoptosis Detection Kit (Beyotime, Shanghai, China) was used for determine the apoptotic cells. Flow cytometry was performed within 30 min.

### RNA Pull-Down

A Pierce™ Magnetic RNA-Protein Pull-Down Kit (cat no. 20164, Thermo Scientific, USA) was used for RNA pull down. Briefly, circYTHDC2 was labeled using Pierce RNA 3′ Desthiobiotinylation Kit (cat No. 20163, Thermo Scientific, USA). Labeled circYTHDC2 was captured using 50 μL of streptavidin magnetic beads in RNA Capture Buffer for 30 min at room temperature. Beads were washed twice in 20 mM Tris (pH 7.5), once in Protein-RNA Binding Buffer and 40 μg of VSMCs extract was added. Samples were incubated for 45 min at 4°C, washed three times with Wash Buffer and eluted after 15 min of incubation at 37°C with Biotin Elution Buffer. RNA pull-down specificity was assessed by Western blotting.

### Western Blotting

Cells were lysed in cold RIPA buffer. A BCA Protein Assay kit (Thermo Fisher Scientific, Rockford, IL, USA) was used for measure protein concentrations. A total of 60 μg of protein was separated on a 10% SDS-PAGE gel and transferred onto polyvinylidene difluoride (PVDF) membranes (Millipore, Billerica, MA, USA). After soaked in 5% non-fat milk for 1 h, the membranes were incubated with primary antibody (anti-YTHDC2, 1:1,000 dilution, cat no. EPR21820-49, Abcam, USA; anti -TET2, 1:1,000 dilution, cat no. EPR20546-135, Abcam, USA; anti-MYOCD, 1:1,000 dilution, cat no. MA5-24103, ThermoFisher Scientific, USA; anti-SRF, 1:1,000 dilution, cat no. 720240, Invitrogen, USA; anti-KLF4, 1:1,000 dilution, cat no. PA5-20897, Invitrogen, USA) incubated overnight at 4°C. After washed with PBS for 3 times, the membranes were incubated with secondary antibody for 1 h at 37°C. The ECL developer is developed in the gel imaging system.

### RNA Binding Protein Immunoprecipitation (RIP)

The RNA immunoprecipitation was performed using the Magna RIPTM RNA-Binding Protein Immunoprecipitation Kit from Millipore, and the experimental steps are performed in accordance with the kit instructions. Briefly, after 48 h transfection, VSMCs cells were harvested, and was lysed with RIP lysis buffer on ice for 10 min. After centrifugation, the supernatant was incubated with 30 μl Protein-A/G magnetic beads (Roche, USA) and antibody (YTHDC2, 10 μg, Abcam) at 4°C overnight. The complex was then centrifuged by immunization and then washed 3 times with washing buffer. The eluted RNA was used for qRT-PCR analysis.

### Luciferase Reporter Gene Assay

The wild type (WT) or mutant (Mut) TET2 cDNA fragments of the target binding site predicted by circYTHDC2 were directly synthesized by Songon Biotech Corporation (Shanghai, China) and inserted into the luciferase report gene pGL3 (Promega) to construct a recombinant vector. A7R5 cells were co-transfected with circYTHDC2 siRNA (50 pmol) and wild-type or mutant TET2 (50 pmol) in a 96-well culture plate (1 × 10^4^ cells per well) using Lipofectamine 3000 according to the manufacturer's instructions. After 48 h of transfection, the luciferase activity was detected by a Thermo Scientific Vanquish system (Thermo Scientific, USA).

### DNA Extraction and Methylation Quantification

Total DNA was extracted from A7R5 cells using a TIANamp Genomic DNA Kit (Tiangen, Beijing, China) according to manufacturer's instructions. DNA concentration was quantified by Nanodrop. Global DNA methylation was quantified using the methylated DNA Quantification (Colorimetric) Kit (ab117128, Abcam, UK) following manufacturer's instructions with plates read at 450 nm on a Bio-Rad microplate reader. Data are represented as percentage of 5-methylcytosine (5-mC) in total DNA.

### Statistics

SPSS 16.0 software was use to carry on statistical analysis to the data. All experimental data were performed 3 separate repetitions, and the data was expressed in the form of mean ± standard deviation (mean ± SD). The obtained data was compared using one-way analysis of variance and Bonferroni's post-correction Student's *t*-test. The difference is statistically significant with *P* < 0.05.

## Results

### The Differentially Expressed circRNA in VSMCs Stimulated by High Glucose

**Figure 1:**
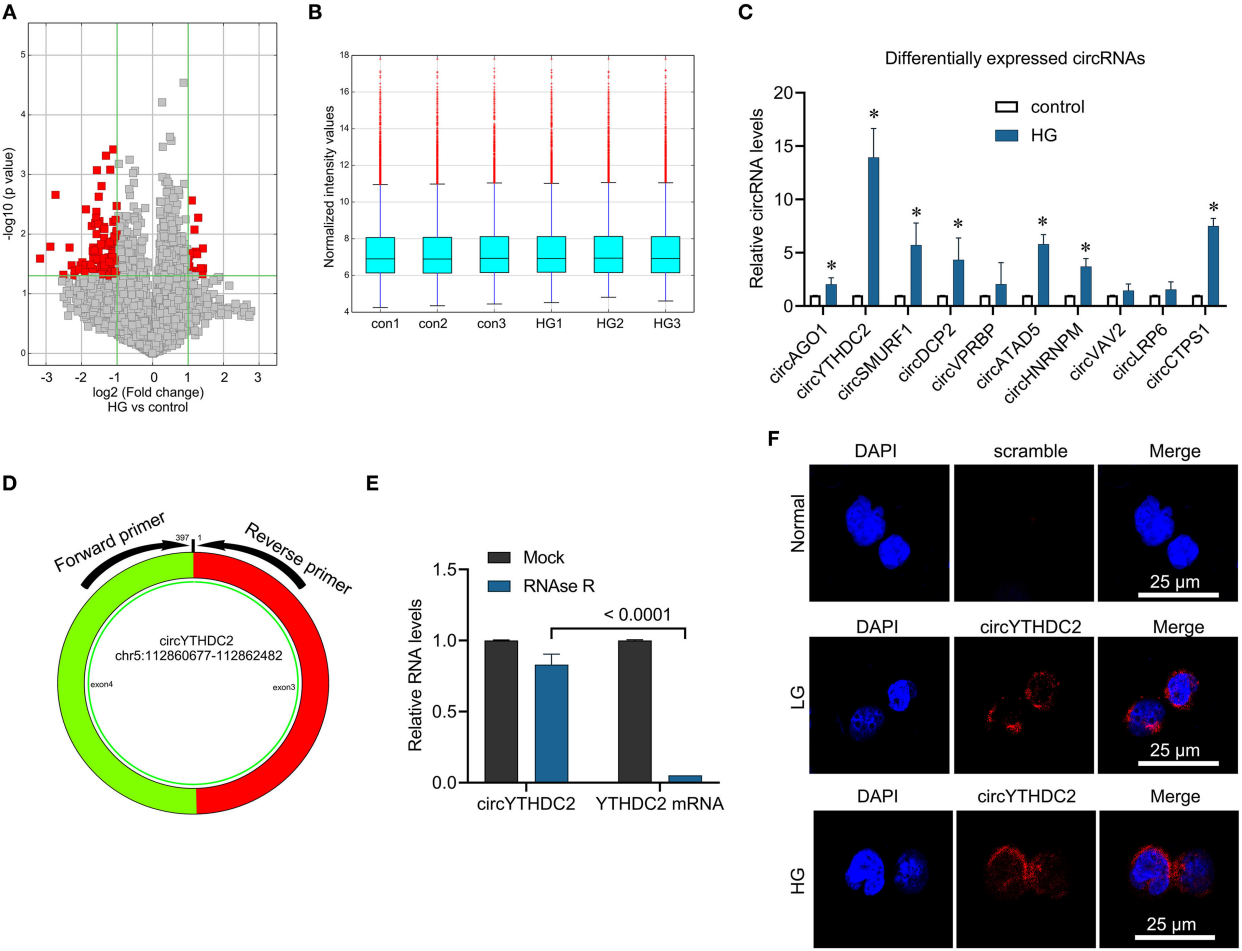
Microarray analysis reveals the aberrantly expressed circRNA in the high glucose-Induced VSMCs. **(A)** Volcano plot presents the differently expressed circular RNAs (fold change > 2, *p*-value < 0.05) in VSMCs treated with high glucose (HG) and control. **(B)** The normalization of differently expressed circular RNAs. **(C)** A7R5 cells were treated with high glucose (25 mmol/L) for 24 h. The cells treated with 5 mmol glucose were used as control. qRT-PCR was performed for verifying the top ten upregulated circRNAs in microarray analysis. *p < 0.05. **(D)** The circYTHDC2 structure and the location of divergent primers for circYTHDC2 was shown. **(E)** qRT–PCR analysis for the expression of circYTHDC2 and YTHDC2 mRNA after treatment with RNase R in A7R5 cells. **(F)** The representative images of RNA fluorescence *in situ* hybridization for circYTHDC2. circYTHDC2 was stained with red, nuclei were stained with DAPI. Bar, 25 μm. Three independent studies were performed and the data were expressed as mean ± standard deviation.

To identify the role of circRNA in high glucose-mediated VSMCs dysfunction, we used high-throughput circRNA microarray to analyze the differential expression of circRNAs in VSMCs stimulated by high glucose. We identified 89 down-regulated circRNAs and 20 up-regulated circRNAs in VSMCs stimulated by high glucose (Figures 1A,B, CircRNA expression profile data was showed in Supplementary Table 2). QPCR verified that high glucose stimulation significantly increased the expression of circAGO2, circYTHDC2, circSMURF1, circDCP2, circATAD5, circHNRNPM, circCTPS1, especially the expression of circYTHDC2 increased by 13 times, while the expression of circVPRBP, circVAV2 and circLRP6 did not change significantly compared with the control group (Figure 1C). Therefore, we chose circYTHDC2 for further analysis. CircYTHDC2 is derived from the back splicing of exons 3 and 4 of YTHDC2 (hg 19, chr5: 112860677–112862482) (Figure 1D). To confirm the circular structure of circYTHDC2, we treated the total RNA with RNase R, and then perform qPCR to measure the expression of circYTHDC2 and YTHDC2. The results showed that RNase R treatment significantly degraded linear YTHDC2 mRNA, but had no significant effect on the expression of circYTHDC2 (Figure 1E), suggesting that circYTHDC2 has a circular structure. In addition, fluorescence *in situ* hybridization (FISH) revealed that circYTHDC2 mainly located in the perinuclear cytoplasm and induced by high glucose stimulation (Figure 1F). These results indicated that circYTHDC2 was highly expressed in VSMCs under high glucose stimulation, and may play an important role in the proliferation and migration of VSMCs.

### CircYTHDC2 Knockdown Inhibits the Proliferation and Migration of VSMCs

**Figure 2:**
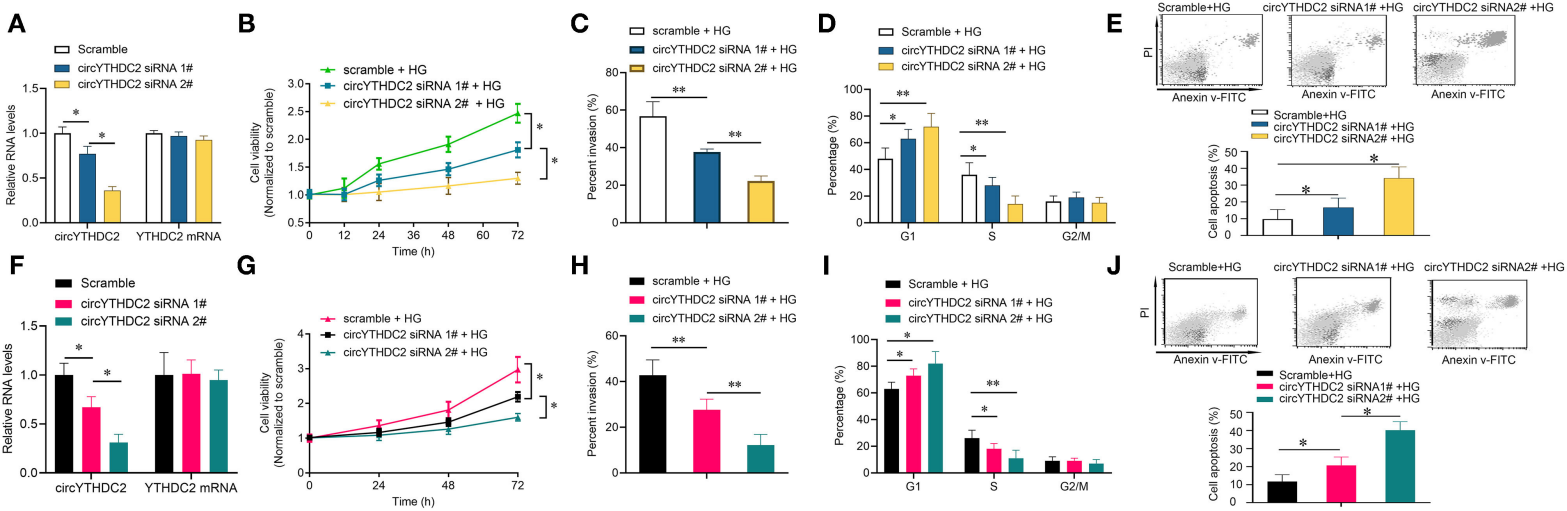
Knockdown of circYTHDC2 inhibits VSMC cell proliferation and invasion under high glucose condition. **(A)** QPCR analysis for the expression of circYTHDC2 and YTHDC2 after circYTHDC2 siRNA transfection in A7R5 cells under high glucose condition. **(B)** CCK-8 assay was performed to determine the cell viability after circYTHDC2 siRNA transfection in A7R5 cells under high glucose condition. **(C)** Transwell assay was performed to determine the cell invasion after circYTHDC2 siRNA transfection in A7R5 cells under high glucose condition. **(D)** Cell cycle assay was performed to determine the cell cycle after circYTHDC2 siRNA transfection in A7R5 cells under high glucose condition. **(E)** Cell apoptosis assay was performed to determine the cell apoptosis after circYTHDC2 siRNA transfection in A7R5 cells under high glucose condition. **(F)** QPCR analysis for the expression of circYTHDC2 and YTHDC2 after circYTHDC2 siRNA transfection in primary VSMCs under high glucose condition. **(G)** CCK-8 assay was performed to determine the cell viability after circYTHDC2 siRNA transfection in primary VSMCs under high glucose condition. **(H)** Transwell assay was performed to determine the cell invasion after circYTHDC2 siRNA transfection in primary VSMCs under high glucose condition. **(I)** Cell cycle assay was performed to determine the cell cycle after circYTHDC2 siRNA transfection in primary VSMCs under high glucose condition. **(J)** Cell apoptosis assay was performed to determine the cell apoptosis after circYTHDC2 siRNA transfection in primary VSMCs under high glucose condition. Three independent studies were performed and the data were expressed as mean ± standard deviation. HG, high glucose. *p < 0.05, **p < 0.01.

To study the role of circYTHDC2 in the proliferation and migration of VSMCs under high glucose, we knocked down the expression of circYTHDC2 in A7R5 cells and primary VSMCs and performed CCK8, Transwell assay, and cell cycle assay. The results showed that circYTHDC2 siRNA transfection significantly reduced the expression of circYTHD2 but not YTHDC2 expression in A7R5 cells (Figure 2A). Cell biological experiments showed that circYTHDC2 knockdown significantly inhibited the proliferation and invasion of A7R5 cells, arrested cell cycle progression from G1 to S phase, and induced cell apoptosis (Figures 2B–E). These results were confirmed in primary VSMCs (Figures 2F–J). Thus, CircYTHDC2 knockdown inhibited the proliferation and migration of VSMCs under high glucose.

### YTHDC2 Positively Regulates circYTHDC2 Through m6A Modification

**Figure 3:**
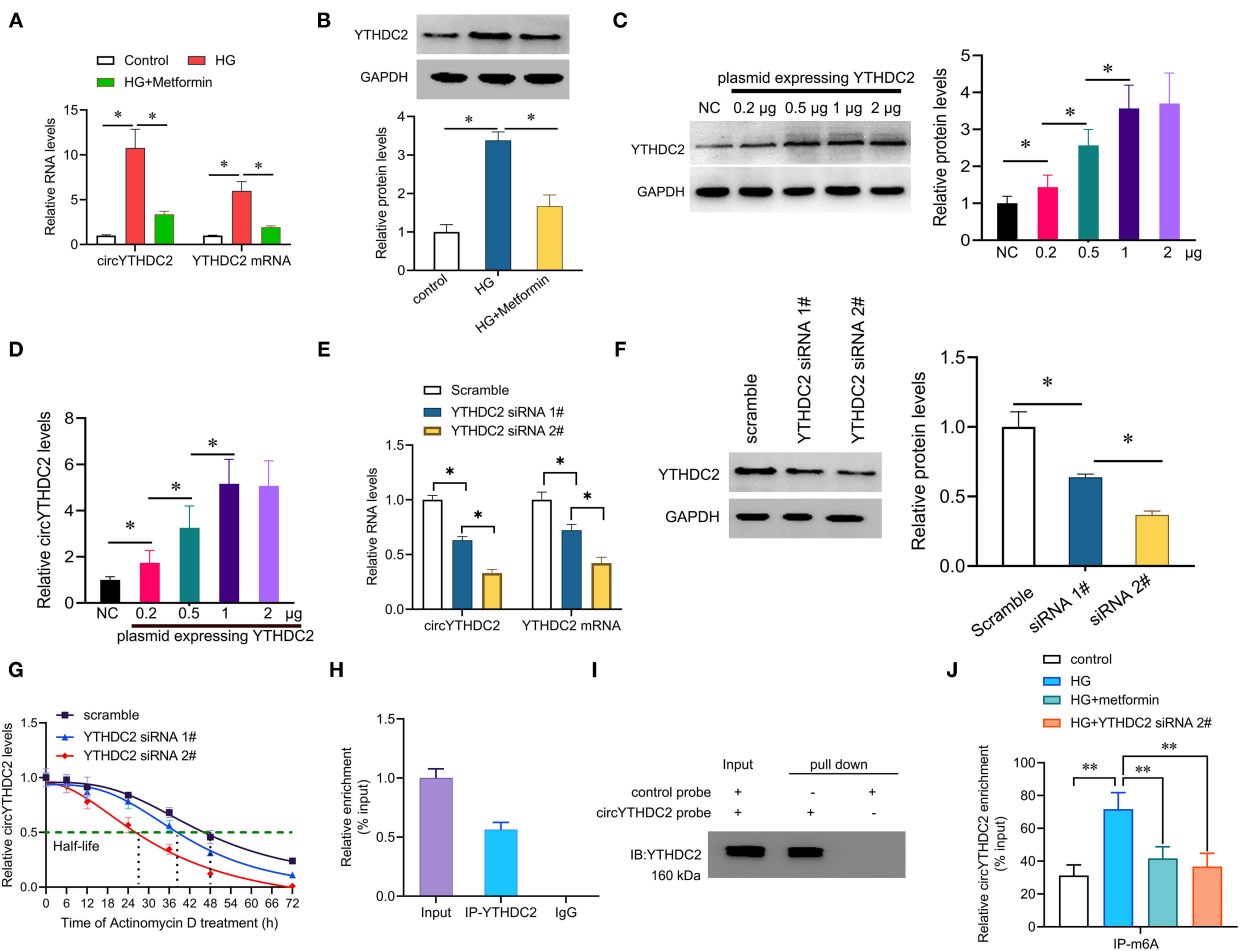
YTHDC2 stabilizes circYTHDC2 by m6A modification. **(A)** qRT–PCR analysis for the expression of circYTHDC2 and YTHDC2 after treatment with high glucose (HG) alone, or together with metformin in A7R5 cells. **(B)** western blot analysis for the expression of YTHDC2 after treatment with high glucose (HG) alone, or together with metformin in A7R5 cells. **(C)** A7R5 cells were transfected with increased plasmids expressing YTHDC2, and western blot was performed to determine the expression of YTHDC2. **(D)** qRT–PCR analysis for the expression of circYTHDC2 after transfection with increased plasmids expressing YTHDC2 in A7R5 cells. **(E)** qRT–PCR analysis for the expression of circYTHDC2 and YTHDC2 mRNA after YTHDC2 siRNA transfection in A7R5 cells. **(F)** western blot was performed to determine the expression of YTHDC2 after YTHDC2 siRNA transfection in A7R5 cells. **(G)** qRT–PCR analysis for the expression of circYTHDC2 after treatment with Actinomycin D at the indicated time points in A7R5 cells transfected with YTHDC2 siRNA. **(H)** RIP assays showing the association of YTHDC2 with circYTHDC2 in A7R5 cells. Relative enrichment representing RNA levels associated with YTHDC2 relative to an input control. IgG antibody served as a control. **(I)** The circYTHDC2-protein complex pulled down by circYTHDC2 junction probe with protein extracts from A7R5 cells. Immunoblot analysis of YTHDC2 after pulldown assay showing its specific association with circYTHDC2. **(J)** RIP assays showing the association of m6A with circYTHDC2 in VSMCs after treatment with high glucose (HG) alone, or together with metformin or YTHDC2 siRNA transfection. Three independent studies were performed and the data were expressed as mean ± standard deviation. *p < 0.05, **p < 0.01.

Next, we wanted to know the relationship between circYTHDC2 and its host gene YTHDC2. Studies have shown that metformin, a first-line hypoglycemic drug, has potential benefits in the treatment of cardiovascular diseases (21). We found that metformin treatment significantly reduced the upregulation of circYTHDC2 that mediated by HG stimulation. Interestingly, metformin also reduced HG-mediated increase in YTHDC2 (Figures 3A,B). In addition, we transfected A7R5 cells with increased amount of plasmid expressing YTHDC2, and found that the expression of circYTHDC2 was upregulated with the increment of YTHDC2 (Figures 3C,D). Silencing YTHDC2 significantly inhibited the expression of circYTHDC2, and reduced the half-life of circYTHDC2 (Figures 3E–G), suggesting that YTHDC2 positively regulates circYTHDC2.

**Figure 4:**
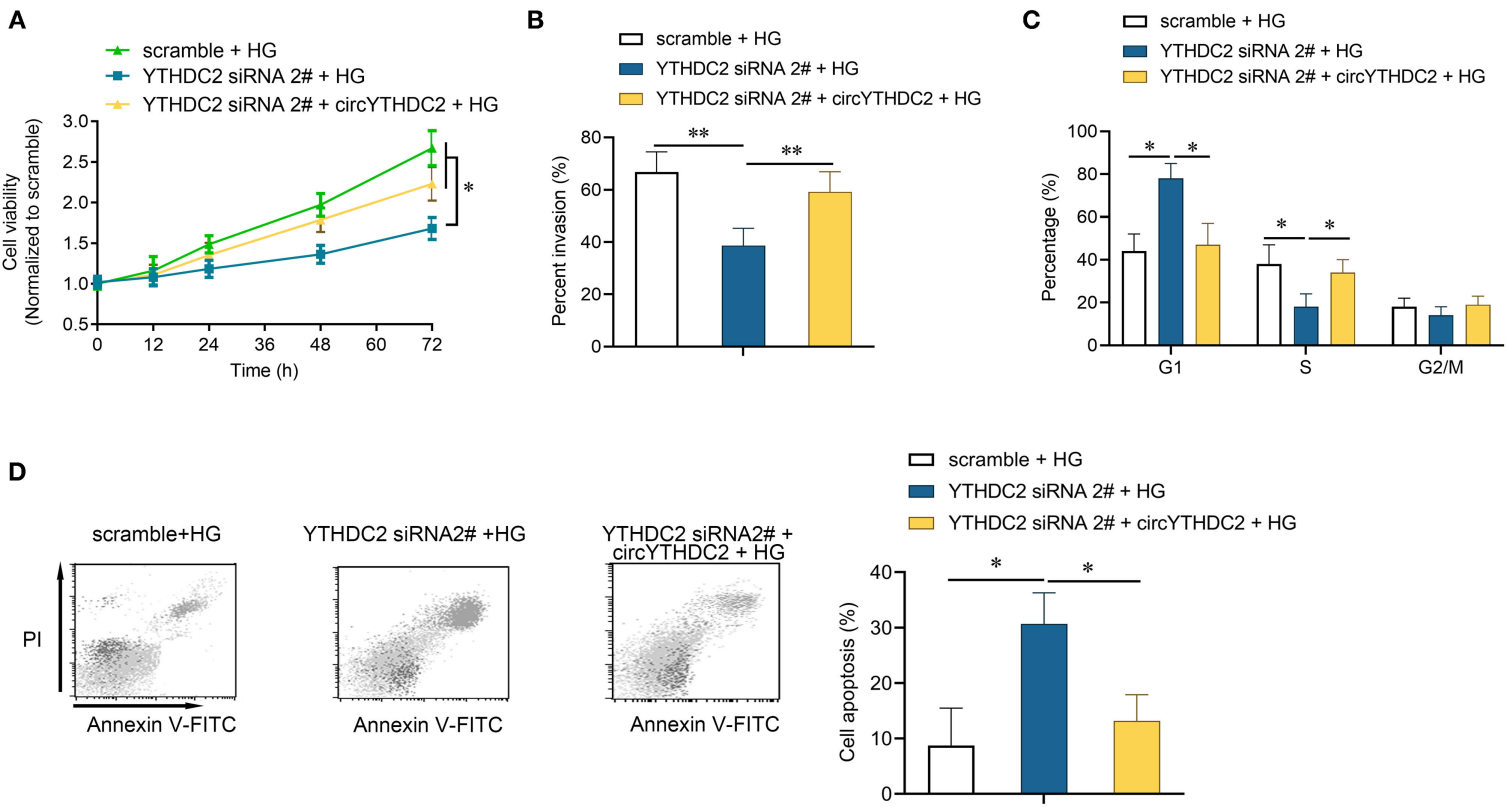
YTHDC2 knockdown inhibits proliferation and migration of VSMCs through circYTHDC2. **(A)** CCK-8 assay was performed to determine the cell viability after YTHDC2 siRNA transfection, or together with circYTHDC2 transfection in A7R5 cells under high glucose condition. **(B)** Transwell assay was performed to determine the cell invasion after YTHDC2 siRNA transfection, or together with circYTHDC2 transfection in A7R5 cells under high glucose condition. **(C)** Cell cycle assay was performed to determine the cell cycle after YTHDC2 siRNA transfection, or together with circYTHDC2 transfection in A7R5 cells under high glucose condition. **(D)** Cell apoptosis assay was performed to determine the cell apoptosis after YTHDC2 siRNA transfection, or together with circYTHDC2 transfection in A7R5 cells under high glucose condition. Three independent studies were performed and the data were expressed as mean ± standard deviation. HG, high glucose. *p < 0.05, **p < 0.01.

We then tested how YTHDC2 regulated circYTHDC2. YTHDC2 is known as a m6A reader and participates in regulating RNA stability. RNA-binding protein immunoprecipitation (RIP) analysis showed that YTHDC2 interacted with circYTHDC2 (Figure 3H). In turn, RNA pull-down experiment also confirmed the interaction between YTHDC2 and circYTHDC2 (Figure 3I). In addition, both metformin treatment and YTHDC2 knockout reduced the m6A modification level of circYTHDC2 (Figure 3J). Further functional studies found that YTHDC2 knockdown inhibited the proliferation and invasion, arrested cell cycle from G1 to S phase in A7R5 cells, and increased apoptotic cells, while overexpression of circYTHDC2 reversed these effects (Figures 4A–D). These results suggest that YTHDC2 positively regulates circYTHDC2 through m6A modification.

### CircYTHDC2 Negatively Regulates TET2

**Figure 5:**
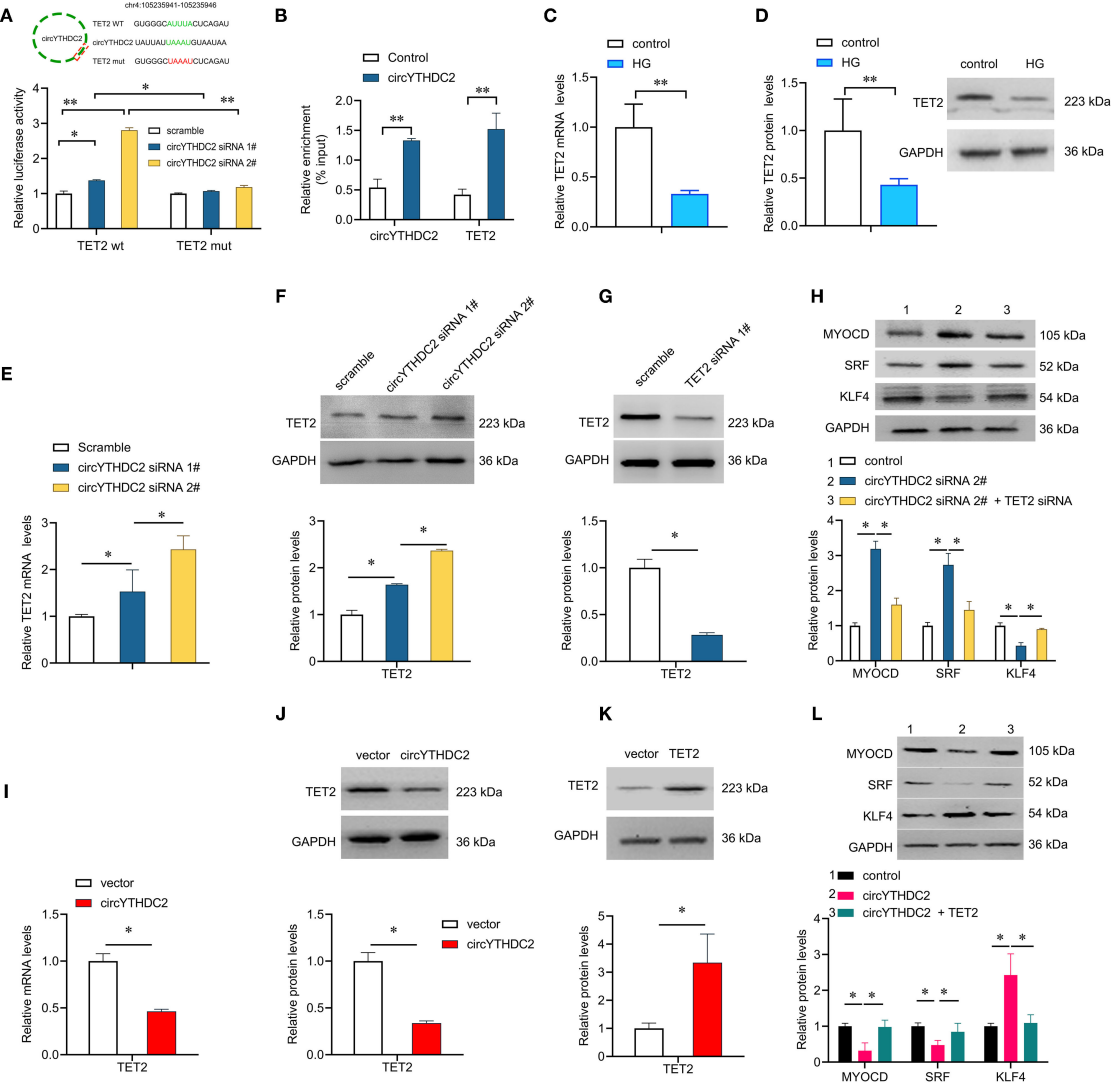
CircYTHDC2 negatively regulates TET2. **(A)** The schematic diagram of TET2-WT or TET2-Mut luciferase reporter gene. The wild type motifs were indicated by green and the mutant motif was indicated by red. A7R5 cells were transfected with TET2-WT or TET2-Mut, combined with circYTHDC2 siRNA. The cells transfected with scramble control were used as control. Luciferase report gene assay were performed to measure the relative luciferase activity. **(B)** Relative enrichment representing TET2 and circYTHDC2 RNA levels associated with circYTHDC2 junction compared to control. **(C)** qRT–PCR analysis for the expression of TET2 after high glucose treatment in A7R5 cells. **(D)** western blotting analysis of TET2 expression after high glucose treatment in A7R5 cells. **(E)** qRT–PCR analysis for the expression of TET2 after circYTHDC2 siRNA transfection in A7R5 cells. **(F)** western blotting analysis of TET2 expression after circYTHDC2 siRNA transfection in A7R5 cells. **(G)** western blotting analysis of TET2 expression after TET2 siRNA transfection in A7R5 cells. **(H)** western blotting analysis for the expression of MYODC, SRF and KLF4 in A7R5 cells after circYTHDC2 siRNA transfection alone, or combined with TET2 siRNA transfection. **(I)** qRT–PCR analysis for the expression of TET2 after circYTHDC2 transfection in A7R5 cells. **(J)** western blotting analysis of TET2 expression after circYTHDC2 transfection in A7R5 cells. **(K)** western blotting analysis of TET2 expression after TET2 transfection in A7R5 cells. **(L)** western blotting analysis for the expression of MYODC, SRF and KLF4 in A7R5 cells after circYTHDC2 transfection alone, or combined with TET2 transfection. Three independent studies were performed and the data were expressed as mean ± standard deviation. HG, high glucose. *p < 0.05, **p < 0.01.

TET2 functions as an upstream regulator of MYOCD/SRF and KLF4, which are key drivers of phenotypic plasticity of VSMC (22). Through AREsite analysis (http://rna.tbi.univie.ac.at/AREsite2) and BLAST analysis, we found that TET2 3′UTR contains multiple AUUUA mRNA unstable motifs, and circYTHDC2 contained the corresponding complementary motif UAAAU, suggesting that circYTHDC2 might regulate the expression of TET2 by binding to the AUUUA motif (Figure 5A). We constructed wild-type TET2 3′UTR (TET2-WT) and mutant TET2 3′UTR (TET2-Mut) luciferase reporter gene, and co-transfected with circYTHDC2 siRNA into A7R5 cells. The results of luciferase report gene assay showed that circYTHDC2 knockdown significantly increased the activity of wild-type TET2-3′UTR luciferase reporter gene, but did not affect the activity of mutant TET2-3′UTR luciferase reporter gene (Figure 5A), confirming the binding of circYTHDC2 on TET2 3′UTR. RNA pull-down analysis also confirmed the interaction between circYTHDC2 and TET2 (Figure 5B). High glucose treatment significantly reduced TET2 expression at mRNA and protein levels (Figures 5C,D). However, we found that that circYTHDC2 knockdown upregulated the expression of TET2, and significantly reduced the 5 mC levels in A7R5 cells (Figures 5E,F; Supplementary Figure 1A). We knocked down TET2 in A7R5 cells (Figure 5G) and detect the downstream target genes of TET2 related to phenotypic plasticity of VSMC. The results showed that circYTHDC2 knockdown significantly up-regulated the expression of contractile-promoting genes myocardin (MYOCD) and serum response factor (SRF), while reduce the expression of dedifferentiation related gene KLF4 (Figure 5H). However, TET2 knockdown reversed the effects of circYTHDC2 knockdown on MYOCD, SRF and KLF4 in VSMCs (Figure 5H). We also performed the reverse experiment to assess whether TET2 could rescue circYTHDC2-mediated VSMCs dedifferentiation. The results revealed that overexpression of circYTHDC2 inhibited TET2 expression (Figures 5I,J), and significantly increased the 5 mC levels in A7R5 cells (Supplementary Figure 1B). In addition, overexpression of TET2 rescued circYTHDC2-mediated SMC dedifferentiation (Figures 5K,L). Thus, these findings suggest that circYTHDC2 negatively regulates TET2 and contributes to phenotype transformation of VSMCs.

## Discussion

In this study, we at first time identified a novel circRNA, circYTHDC2. We revealed that circYTHDC2 was induced by high glucose stimulation in VSMCs. Knockdown of circYTHDC2 inhibited the proliferation and invasion, and arrested cell cycle of VSMCs.

In recent years, circRNAs have been reported to be associated with human diseases, such as coronary atherosclerotic heart disease (7). For example, Mao et al. found that circSATB2 promoted cell proliferation and migration of VSMCs by increasing the expression of STIM1 (9). Hall et al. reported that circLrp6 regulated miR-145-mediated vascular smooth muscle cell migration, proliferation, and differentiation (23). These studies indicate that circRNAs play an important role in VSMCs dysfunction, and are expected to become a new target for the diagnosis, treatment, and prognosis of cardiovascular disease. In this study, we found that YTHDC2 modified the stability of circYTHDC2 through m6A modification. As a m6A binding protein and m6A reader, YTHDC2 participates in various biological processes (24), and is also involved in the occurrence of tumors and other diseases (17, 25). However, the role of YTHDC2 in VSMCs dysfunction remain unclear. We here reported that YTHDC2 enhanced the proliferation and invasion of VSMCs by binding to circYTHDC2 and promoting the expression of circYTHDC2. Kretschmer et al. showed that YTHDC2 was mainly enriched in the perinuclear region, and bound to ribosomes to improve translation efficiency (16, 26). For example, He et al. found that YTHDC2 bound to insulin-like growth factor 1 receptor (IGF1R) mRNA, promoted the translation initiation of the transcript, and then activated the IGF1R-AKT/S6 signaling pathway (27). In this study, the FISH results showed that circYTHDC2 was mainly enriched in the perinuclear region, and YTHDC2 enhanced the stability of circYTHDC2 through m6A modification, suggesting that YTHDC2 functions as a gene activator under this context.

We further studied the downstream effector of circYTHDC2 and found that Ten-eleven translocation 2 (TET2) was negatively regulated by circYTHDC2. Ten-eleven translocation protein family includes 3 members: TET1, TET2 and TET3. TET proteins oxidize 5-methylcytosine (5 mC) to 5-hydroxymethylcytosine (5 hmC), thereby mediating active DNA demethylation (28). We here found that circYTHDC2 knockdown significantly reduced the 5 mC levels in A7R5 cells, suggesting that circYTHCD2 might regulate DNA methylation status through TET2. TET2 is a master regulator of smooth muscle cell plasticity by upregulating the expression of the contractile-promoting gene myocardin (MYOCD) and serum response factor (SRF), whereas downregulating the dedifferentiation-related gene KLF4 (29). TET2 is closely associated with the phenotypic transformation of VSMC (22). Under the stimulation of injury or inflammatory factors such as high glucose, VSMCs transform from a differentiated “contraction type” to a dedifferentiated “synthetic type.” In this study, we focused on the role of circYTHDC2 on VSMC plasticity, and found that circYTHDC2 knockdown can significantly up-regulate the expression of contractile-promoting genes MYOCD and SRF, while reduce the expression of dedifferentiation related gene KLF4 by inducing TET2, suggesting that circYTHDC2 promotes dedifferentiated “synthetic type” transformation of VSMC under high glucose condition.

## Conclusion

In this study, we identified a new circRNA, circYTHDC2, and found that circYTHDC2 was significantly induced by high glucose stimulation in VSMCs. Knockout of circYTHDC2 inhibited the proliferation and invasion of VSMCs. Further mechanism studies found that YTHDC2 stabilizes circYTHDC2 through m6A modification, and circYTHDC2 negatively regulates TET2 to promoting “synthetic type” transformation of VSMC under high glucose condition. YTHDC2/circYTHDC/TET2 axis may become a potential therapeutic target for cardiovascular disease.

## Data Availability Statement

The original contributions presented in the study are included in the article/Supplementary Material, further inquiries can be directed to the corresponding author/s.

## Ethics Statement

The animal study was reviewed and approved by the Ethics Committee for Animal Research of The People's Hospital of Guangxi Zhuang Autonomous Region.

## Author Contributions

JY conceived the study and participated in the study design, performance, coordination, and manuscript writing. YL, LZ, YX, ZL, and JG performed the research. All authors have read and approved the final manuscript.

## Conflict of Interest

The authors declare that the research was conducted in the absence of any commercial or financial relationships that could be construed as a potential conflict of interest.

## Publisher's Note

All claims expressed in this article are solely those of the authors and do not necessarily represent those of their affiliated organizations, or those of the publisher, the editors and the reviewers. Any product that may be evaluated in this article, or claim that may be made by its manufacturer, is not guaranteed or endorsed by the publisher.

## Supplementary Material

Supplementary Figure 1 CircYTHDC2 enhances DNA methylation. Total DNA methylation in A7R5 cells transfected with circYTHDC2 siRNAs **(A)** or circYTHDC2 expressing plasmids **(B)** were determined by measuring 5-methylcytosine (5-mC). ^*^*p* < 0.05.

Supplementary Table 1 Primers.
