# Supplementary figures and images for: YTHDC2-Mediated circYTHDC2 N6-Methyladenosine Modification Promotes Vascular Smooth Muscle Cells Dysfunction Through Inhibiting Ten-Eleven Translocation 2
Source: Front Cardiovasc Med. 2021 Oct 1;8:686293. doi: 10.3389/fcvm.2021.686293 (PMC8517116; doi:10.3389/fcvm.2021.686293)

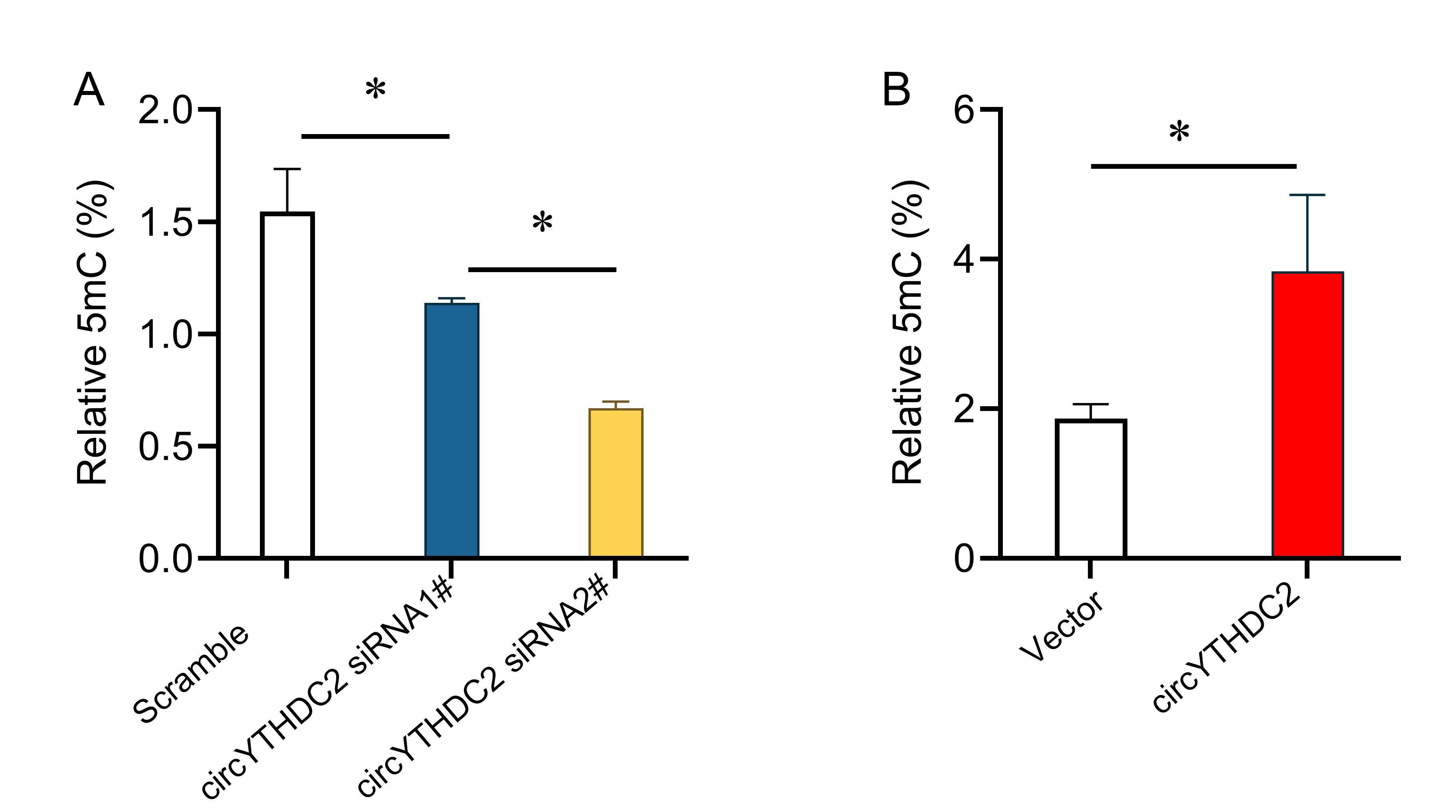

Supplement: Supplementary Figure 1 — CircYTHDC2 enhances DNA methylation. Total DNA methylation in A7R5 cells transfected with circYTHDC2 siRNAs (A) or circYTHDC2 expressing plasmids (B) were determined by measuring 5-methylcytosine (5-mC). *p < 0.05. [file Image_1.TIF]
