# Supplementary material for: YTHDC2-Mediated circYTHDC2 N6-Methyladenosine Modification Promotes Vascular Smooth Muscle Cells Dysfunction Through Inhibiting Ten-Eleven Translocation 2
Source: Front Cardiovasc Med. 2021 Oct 1;8:686293. doi: 10.3389/fcvm.2021.686293 (PMC8517116; doi:10.3389/fcvm.2021.686293)
Supplement: Supplementary Table 1 — Primers. [file Data_Sheet_1.PDF]

Supplementary Table1. primers

Primer name	qPCR primer sequence (5'→3')
circAGO1-Forward	ATCTGCCCTGCCTACAAGTT
circAGO1-Reverse	ATTCGATCCTTCCCTTCCCC
circYTHDC2 divergent-Forward	AGGAGAAACTGGGTCTGGAA
circYTHDC2 divergent-Reverse	CAGCTTCAACTGCAAACACA
circSMURF1-Forward	CTTCCTGCCCAGAGATACGA
circSMURF1-Reverse	TAAACTTGGCCCTGGACTGT
circDCP2-Forward	GGCGATTCCTCAGACAGTGA
circDCP2-Reverse	TGTACAAACGAGCAAGCTGG
circVPRBP-Forward	TCACGGGAGCTGATGATGTT
circVPRBP-Reverse	TCACGGGAGCTGATGATGTT
circATAD5-Forward	CAGCGCAGTGGTAGACAAAT
circATAD5-Reverse	TGCCACCCGAGAAATCTTCA
circHNRNPM-Forward	TGGAGCTCTTAATGGACGCT
circHNRNPM-Reverse	TTCTGAGCAGGTCGTTCTCC
circVAV2-Forward	AGACCAAAGATTGACGGGGA
circVAV2-Reverse	TGCACCTCCACCTTGATGAT
circLRP6-Forward	GCATGTGATTGGCTTGGAGA
circLRP6-Reverse	CCTCCAAGCCTCCAACTACA
circCTPS1-Forward	CAATGTTCTGCCATGTTGAGC
circCTPS1-Reverse	AGCCCAAGTCCTCTAAGTTCC
YTHDC2-Forward	GAGGCCTTTCTGGTGACCTC
YTHDC2-Reverse	TTGTTGAGTCGCCCACTTGT
β-actin-Forward	GGGAAATCGTGCGTGACATTAAG
β-actin-Reverse	TGTGTTGGCGTACAGGTCTTTG
